# Association of sleep traits with male fertility: a two-sample Mendelian randomization study

**DOI:** 10.3389/fgene.2024.1353438

**Published:** 2024-02-22

**Authors:** Shikuan Lu, Ziyang Ma, Wanzhen Zhou, Hongsen Zeng, Jian Ma, Hang Deng, Peihai Zhang

**Affiliations:** ^1^ Hospital of Chengdu University of Traditional Chinese Medicine, Chengdu, China; ^2^ Chengdu University of Traditional Chinese Medicine, Chengdu, China

**Keywords:** male sterility, sleep, Mendelian randomization, causal relationship, gene

## Abstract

**Background:** Previous observational studies have investigated the association between sleep-related traits and male fertility; however, conclusive evidence of a causal connection is lacking. This study aimed to explore the causal relationship between sleep and male fertility using Mendelian randomisation.

**Methods:** Eight sleep-related traits (chronotype, sleep duration, insomnia, snoring, dozing, daytime nap, oversleeping, and undersleeping) and three descriptors representing male fertility (male infertility, abnormal sperm, and bioavailable testosterone levels) were selected from published Genome-Wide Association Studies. The causal relationship between sleep-related traits and male fertility was evaluated using multiple methods, including inverse variance weighting (IVW), weighted median, Mendelian randomisation-Egger, weighted model, and simple model through two-sample Mendelian randomisation analysis. Mendelian randomisation-Egger regression was used to assess pleiotropy, Cochrane’s Q test was employed to detect heterogeneity, and a leave-one-out sensitivity analysis was conducted.

**Results:** Genetically-predicted chronotype (IVW,OR = 1.07; 95%CL = 1.04–1.12; *p* = 0.0002) was suggestively associated with bioavailable testosterone levels. However, using the IVW method, we found no evidence of a causal association between other sleep traits and male fertility.

**Conclusion:** This study found that chronotype affects testosterone secretion levels. However, further studies are needed to explain this mechanism.

## 1 Introduction

Infertility is a disease of the male or female reproductive system defined by the failure to achieve a pregnancy after 12 months or more of regular unprotected sexual intercourse (World Health Organization, 2018). Infertility affects millions of people—and has an impact on their families and communities. The World Health Organization estimates that approximately one in every six people of reproductive age worldwide experience infertility in their lifetime, in half of which the man is infertile (World Health Organization, 2023). Male infertility has become a significant factor affecting the global population development (Agarwal et al., 2015). Many genetic and lifestyle factors have been implicated in male infertility.To reduce the social and public health burden of male infertility, it is crucial to identify preventive causes, particularly modifiable risk factors (Sharma et al., 2013).

The hectic lifestyles humans have been obliged to follow in recent decades have affected sleep quality, causing an increase in sleep disorders (Grandner, 2017).Sleep disorders and male infertility are common global public health issues in contemporary society. Sleep is crucial for overall health, and good sleep is beneficial for health (Hirshkowitz et al., 2015). Sleep disorders have complex phenotypes driven by genetic and lifestyle factors that contribute to various health problems. In the past few decades, there has been an increasing interest in exploring the extent to which disrupted sleep patterns affect adverse health outcomes (Lane et al., 2017). Growing evidence suggests that sleep quality has a significant impact on human health, and insufficient sleep increases the risk of conditions such as hypertension, diabetes mellitus type 2 (T2DM), cardiovascular diseases, depression, cancer, and male infertility (Hvidt et al., 2020). Recent studies have shown that sleep disorders may also be one of the important factors leading to infertility. Sleep quality can affect a person’s mental state, brain function, metabolism and hormone levels, which in turn affect reproductive function and lead to infertility (Auger et al., 2021).The extent to which sleep duration affects male fertility is not yet clear; however, ecological data suggest a correlation over the past few decades between an increase in sleep deprivation and a decline in sperm counts among Western men (Ford et al., 2015; Levine et al., 2017). Several studies conducted abroad have found a U-shaped relationship between sleep duration and male fertility, in which both insufficient and excessive sleep are associated with poor fertility outcomes (Jensen et al., 2013; Wise et al., 2018; Wang et al., 2021). Furthermore, multiple studies have indicated that shift work disrupts the wake/sleep cycle and leads to circadian rhythm disruption, which, in turn, contributes to cardiovascular diseases, metabolic disorders, and male infertility (Torquati et al., 2018; Demirkol et al., 2021). A previous study revealed that sperm density, total motile count, and hormone levels were lower in shift workers than in non-shift workers (Deng et al., 2018). Similarly, a prospective cohort study confirmed that shift work significantly increases the risk of male infertility (El-Helaly et al., 2010). The impact of sleep on fertility is often overlooked when studying male infertility. There is a significant lack of research on the impact of sleep disorders on reproduction (Caetano et al., 2021). Despite several studies indicating correlations between sleep-related traits and male fertility, previous observational studies may have been subject to bias due to reverse causality and confounding factors. Therefore, there is no consensus regarding the causal relationship between sleep-related traits and male fertility.

Mendelian randomisation (MR) analysis is an emerging epidemiological research method considered an ideal tool for optimising the design of subsequent randomised trials (Ference et al., 2021). By including exposure-associated genetic variants of interest as instrumental variables, MR can avoid unmeasured confounding factors in observational studies and examine the causal relationship between potentially modifiable risk factors and health outcomes (Davies et al., 2018). In addition, the effect of genetic variation on exposure is present since conception, indicating that MR can assess the effect of lifetime exposure on outcome risk (Hemani et al., 2018). Therefore, it is particularly important to use MR analysis to infer the causal relationships between sleep-related traits and male fertility. In this study, we used a two-sample Mendelian randomisation design to infer the causal associations between eight sleep traits and male fertility.

## 2 Methods

### 2.1 Study design

**FIGURE 1:**
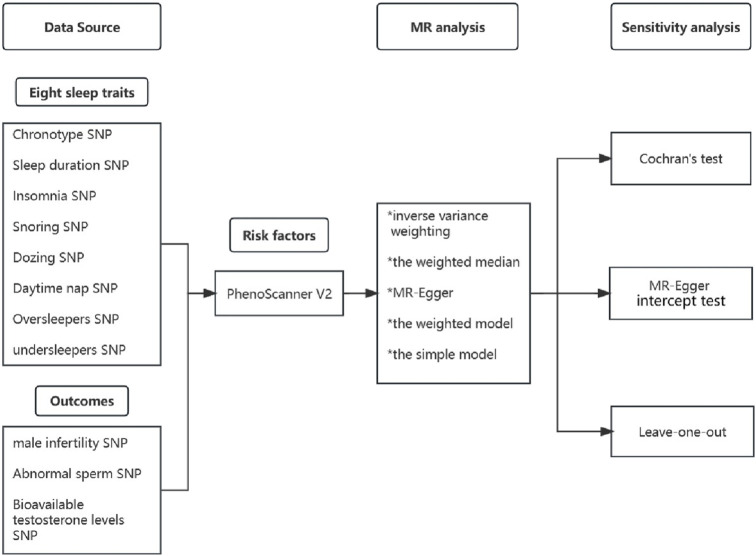
Overview of study design.

Figure 1 presents an overview of the study design. In this study, we used various sleep-related traits, including chronotype, sleep duration, insomnia, snoring, dozing, daytime napping, oversleeping, and undersleeping. Male infertility, abnormal sperm, and bioavailable testosterone levels were used to define male fertility. Mendelian randomisation was used to examine the causal relationship between sleep traits and male fertility.

### 2.2 Data sources

Our exposure and outcome data were obtained from the Ieu Open Genome-Wide Association Study (GWAS) project database (https://gwas.mrcieu.ac.uk/), which is a large cohort study that has collected more than 500,000 people from all over the UK. All the participants were of European ancestry. These included the chronotype (*n* = 413,343), sleep duration (*n* = 460,099), insomnia (*n* = 462,341), snoring (*n* = 430,438), dozing (*n* = 460,913), sleep duration (*n* = 460,099). daytime nap (*n* = 452,633), oversleeping (*n* = 91,306), undersleeping (*n* = 110,188), male infertility (*n* = 72,799), abnormal sperm (*n* = 209,006), and bioavailable testosterone levels (*n* = 382,988).

Because the study was based on published data, no ethical approval or informed consent was required.

### 2.3 Selection of instrumental variables

Mendelian randomisation should satisfy three core assumptions to obtain unbiased estimates: 1) genetic variants (instrumental variables) are strongly associated with sleep traits (exposure); 2) genetic variants do not share common causes (potential confounders) with male fertility-related indicators (outcomes); 3) genetic variation affects male fertility-related indicators (outcomes) only through its effect on sleep-related traits (exposure) (Bowden et al., 2015).

In this study, to identify the best instrumental variable for sleep, we used the following steps to select IVs and ensure that genetic instruments were associated with sleep. Single nucleotide polymorphisms (SNP) that were strongly associated with sleep traits were extracted from the GWAS project database, and p < 5E-8 was used as the main screening condition. For oversleepers and undersleepers, a more relaxed threshold was used (p < 5E-5) to select more SNPs. Second, linkage disequilibrium (LD) SNPs were eliminated (r2 0.001, clumping window = 10,000 kb) to ensure exposure instrument independence, and to avoid bias due to weak IV. We used the F statistic to measure the strength of the IVs. A weak IV was defined as an F-statistic less than 10, and all weak instrumental variables were excluded. We then used the PhenoScanner V2 website (http://www. phenoscanner. medschl. cam. ac = /) to exclude single nucleotide polymorphisms (SNPs) that are potentially confounding factors and related to the outcome (male fertility description) to eliminate the possibility of genetic pleiotropy. After a series of rigorous screenings, the remaining SNPS were considered eligible for IV.

### 2.4 Statistical analysis

We used five methods: inverse variance weighting (IVW), weighted median (WM), MR-Egger, weighted model, and a simple model, to assess the causal relationship between exposure (sleep-related traits) and outcome (male fertility), with inverse variance weighting (IVW) being the primary statistical analysis method. In the IVW model, *p* < 0.05 is considered statistically significant. The remaining four methods were used for complementary analyses. When heterogeneity was significant, the weighted median method was used as an auxiliary approach. The MR-Egger regression method was used to assess pleiotropy using the intercept test. The weighted model then estimates individual proportions based on SNPs, groups the SNPs based on their similarity, calculates the inverse variance-weighted sum for each group of SNPs, and derives the causal estimate based on the group with the highest weighted sum (Hwang et al., 2019). Finally, if at least 50% of the IVs are valid, the simple median provides a consistent estimate of the causal effect (Bowden et al., 2016).

We used several sensitivity analyses to examine and correct causal estimates. First, we performed a heterogeneity test, which can indicate the reliability of the MR estimates, where Cochrane’s Q value can indicate heterogeneity among the selected IVs. We used the Egger regression intercept to estimate the magnitude of horizontal pleiotropy, which could provide further insights into whether SNPs influence male fertility through sleep traits. Finally, leave-one-out sensitivity analyses were performed to confirm that the causality was not driven by a single IV.

The results are reported as odds ratios (OR) along with their corresponding 95% confidence intervals (CI) and *p*-values, with statistical significance considered at *p* < 0.05. All analyses were conducted using R statistical software version 4.3.1 and the R package “TwoSampleMR”.

## 3 Results

### 3.1 Sleep-related characteristics and male infertility

According to IVW analysis, we did not find that a genetically-predicted chronotype (OR = 0.88; 95% CL = 0.42–1.83; *p* = 0.725), sleep duration (OR = 0.99; 95% CL = 0.26–3.77; *p* = 0.994), insomnia (OR = 0.34; 95% CL = 0.05–2.49; *p* = 0.290), snoring (OR = 0.53; 95% CL = 0.03–9.40; *p* = 0.667), dozing (OR = 3.62; 95% CL = 0.19–70.21; *p* = 0.395), daytime nap (OR = 2.64; 95% CL = 0.67–10.40; *p* = 0.164), oversleeping (OR = 1.55; 95% CL = 0.25–9.58; *p* = 0.635), or undersleeping (OR = 4.38; 95% CL = 0.44–43.22; *p* = 0.206) had a causal connection to a diagnosis of male infertility (Table 1). The other four analysis methods revealed consistent estimates (Supplementary Figure S1). However, several sensitivity analyses did not detect heterogeneity or horizontal pleiotropy (Table 2). Among them, the heterogeneity of the time type was high; therefore, IVW meta-analysis under the random-effects model was used to mitigate the impact of heterogeneity.

**TABLE 1:** Associations of genetically-predicted sleep characteristics with male infertility.

Exposure	No, of Snps	OR (95%CI)	P
Chronotype	147	0.88 (0.42–1.83)	0.725
Sleep duration	64	0.99 (0.26–3.77)	0.994
Insomnia	38	0.34 (0.05–2.49)	0.290
Snoring	39	0.53 (0.03–9.40)	0.667
Dozing	30	3.62 (0.19–70.21)	0.395
Daytime nap	93	2.64 (0.67–10.40)	0.164
oversleepers	31	1.55 (0.25–9.58)	0.635
undersleepers	25	4.38 (0.44–43.22)	0.206

SNP, single-nucleotide polymorphism; CI, confidence interval; OR, odds ratio.

**TABLE 2:** Sensitivity analysis of sleep characteristics and male infertility.

Exposure	Pleiotropy		Heterogeneity	
	Intercept	p	Q	p
Chronotype	0.001	0.65	189	0.01
Sleep duration	0.015	0.62	53	0.82
Insomnia	0.004	0.9	49	0.08
Snoring	−0.052	0.34	43	0.28
Dozing	0.015	0.77	22	0.83
Daytime nap	0.001	0.95	79	0.83
oversleepers	−0.001	0.73	34	0.27
undersleepers	0.028	0.42	22	0.62

### 3.2 Sleep-related characteristics and sperm abnormalities

According to the results of two-sample MR analysis, chronotype (IVW,OR = 0.97; 95% CL = 0.55–1.69; *p* = 0.907), sleep duration (IVW,OR = 0.89; 95% CL = 0.26–3.03; *p* = 0.854), insomnia (IVW,OR = 1.07; 95% CL = 0.24–4.81; *p* = 0.933), and snoring (IVW,OR = 8.49; 95% CL = 0.81–8.89 E+01; *p* = 0.074) did not show a causal relationship with the risk of abnormal sperm (Supplementary Figure S4). Similarly, we did not find a genetically-predicted risk from dozing (IVW,OR = 0.79; 95% CL = 6.04 E-02–10.27; *p* = 0.855), daytime nap (IVW,OR = 0.83; 95% CL = 0.25–2.72; *p* = 0.762), oversleeping (IVW,OR = 2.73; 95% CL = 0.57–13.08; *p* = 0.208), undersleeping (IVW,OR = 0.50; 95% CL = 0.07–3.78; *p* = 0.503) and the risk of abnormal sperm (Table 3). The other four analytical methods yielded consistent estimates (Supplementary Figure S2). The Cochrane Q statistic showed no significant heterogeneity and the MR-Egger regression results showed no horizontal pleiotropy (Table 4).

**TABLE 3:** Associations of genetically-predicted sleep characteristics with abnormal sperm.

Exposure	No, of Snps	OR (95%CI)	P
Chronotype	147	0.97 (0.55–1.69)	0.907
Sleep duration	64	0.89 (0.26–3.03)	0.854
Insomnia	38	1.07 (0.24–4.81)	0.933
Snoring	39	8.49 (0.81–8.89E+01)	0.074
Dozing	30	0.79 (6.04E-02–10.27)	0.855
Daytime nap	93	0.83 (0.25–2.72)	0.762
oversleepers	31	2.73 (0.57–13.08)	0.208
undersleepers	25	0.50 (0.07–3.78)	0.503

SNP, single-nucleotide polymorphism; CI, confidence interval; OR, odds ratio.

**TABLE 4:** Sensitivity analysis of sleep characteristics and abnormal sperm.

Exposure	Pleiotropy		Heterogeneity	
	Intercept	p	Q	p
Chronotype	0.024	0.1	146	0.48
Sleep duration	0.043	0.14	70	0.24
Insomnia	0.008	0.77	27	0.88
Snoring	−0.057	0.21	30	0.82
Dozing	0.036	0.43	26	0.64
Daytime nap	0.015	0.44	92	0.49
oversleepers	−0.001	0.94	31	0.41
undersleepers	−0.006	0.84	22	0.64

### 3.3 Sleep-related characteristics and bioavailable testosterone levels

**FIGURE 2:**
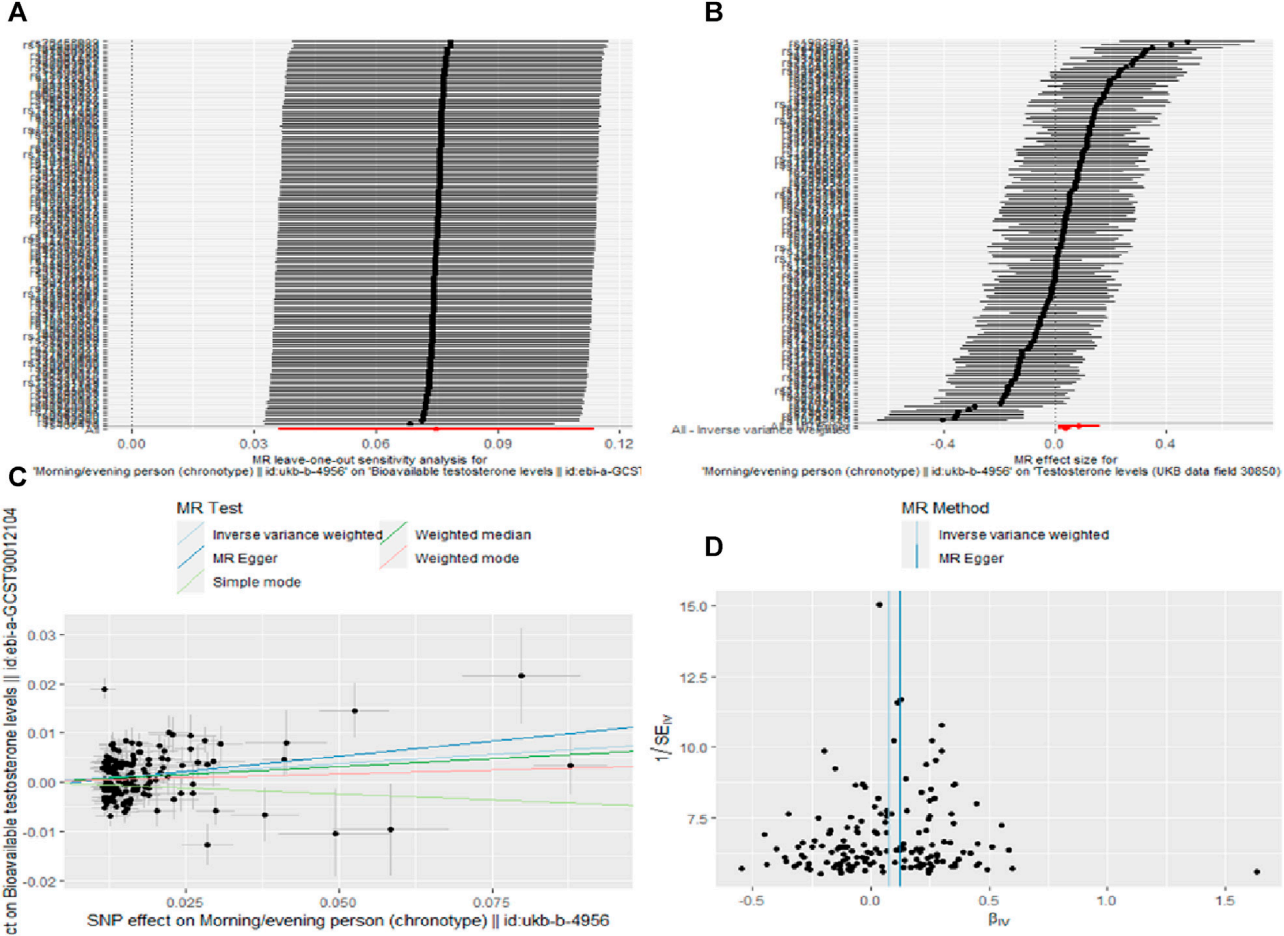
Sensitivity analysis **(A)**, forest plot **(B)**, scatter plot **(C)** and funnel plot **(D)** the causal effect of Chronotype on Bioavailable testosterone levels.

This study identified a set of causal relationships. A causal relationship was found between chronotype and bioavailable testosterone levels (IVW,OR = 1.07; 95% CL = 1.04–1.12; *p* = 0.0002). However, we did not find a causal relationship between genetically-predicted sleep duration (OR = 1.04; 95% CL = 0.95–1.14; *p* = 0.434), insomnia (OR = 1.09; 95% CL = 0.95–1.26; *p* = 0.229), snoring (OR = 0.89; 95% CL = 0.67–1.20; *p* = 0.446), dozing (OR = 0.97; 95% CL = 0.79–1.19; *p* = 0.773), daytime nap (OR = 1.09; 95% CL = 1.00–1.19; *p* = 0.052), oversleeping (OR = 1.07; 95%CL = 0.96–1.19; *p* = 0.211), undersleeping (OR = 1.03; 95% CL = 0.91–1.17; *p* = 0.629) and bioavailable testosterone levels (Table 5). The other four analyses yielded consistent results (Supplementary Figure S3). Heterogeneity (*p* < 0.05) was observed in chronotypes, sleep duration, insomnia, snoring, dozing, daytime nap, and undersleeping through sensitivity analysis (Table 6). Therefore, we used IVW meta-analysis under the random-effects model to reduce the impact of heterogeneity. No pleiotropy was detected in the sensitivity analyses. For positive results, the leave-one-out analyses demonstrated the consistency of the results (Figure 2A), and the remaining visualisations of the results of MR analysis are shown in Supplementary Figure S8.

**TABLE 5:** Associations of genetically-predicted sleep characteristics with bioavailable testosterone levels.

Exposure	No, of Snps	OR (95%CI)	P
Chronotype	155	1.07 (1.04–1.12)	**0.0002**
Sleep duration	69	1.04 (0.95–1.14)	0.434
Insomnia	39	1.09 (0.95–1.26)	0.229
Snoring	42	0.89 (0.67–1.20)	0.446
Dozing	31	0.97 (0.79–1.19)	0.773
Daytime nap	105	1.09 (1.00–1.19)	0.052
oversleepers	35	1.07 (0.96–1.19)	0.211
undersleepers	28	1.03 (0.91–1.17)	0.629

SNP, single-nucleotide polymorphism; CI, confidence interval; OR, odds ratio. Bold values represent positive results.

**TABLE 6:** Sensitivity analysis of the sleep characteristics and bioavailable testosterone levels.

Exposure	Pleiotropy		Heterogeneity	
	Intercept	p	Q	P
Chronotype	−0.0008	0.43	450	8.45E-31
Sleep duration	−0.001	0.58	292	1.38E-29
Insomnia	0.003	0.21	204	1.60E-24
Snoring	−0.003	0.62	401	1.46E-60
Dozing	0.003	0.36	114	9.67E-12
Daytime nap	0.002	0.12	376	2.05E-32
oversleepers	−0.0002	0.86	37	0.35
undersleepers	0.0002	0.91	61	0.0002

## 4 Discussion

In this study, we used the British Bank of Biological Data Sets Mendelian randomisation analysis, a systematic evaluation of eight sleep-related traits, and male fertility (male infertility, abnormal sperm, and bioavailable testosterone levels). We found a causal relationship between genetically-predicted chronotype and bioavailable testosterone levels. However, we found no association between the other seven sleep-related characteristics and male fertility.

The circadian rhythm, generated by a core set of clock genes, is an intrinsic timing system that synchronises an organism’s cellular, behavioural, and physiological processes with the Earth’s rotation, including sleep-wake preferences, body temperature, hormone secretion, food intake, and cognitive and physical performance. Individual differences in sleep-wake cycles lead to the emergence of distinct behavioural phenotypes called chronotypes. Under normal conditions, the endogenous rhythm of the sleep-wake cycle is synchronised with alterations in the circadian cycle (Roenneberg et al., 2003; Sack et al., 2007; Montaruli et al., 2021). Disruption of circadian rhythms can lead to various pathological disorders and diseases. Testosterone is essential for maintaining spermatogenesis (Smith and Walker, 2014). Studies have found that the rhythm of testosterone production is circadian in normal men and begins to rise during sleep onset and peaks during the first REM sleep (Luboshitzky et al., 2001). Meanwhile, sleep deprivation in the second half of the night has been shown to significantly lower testosterone levels in the morning (Schmid et al., 2012). In addition, relevant animal studies have shown that the steroidogenic-related genes which are responsible for testosterone production in Leydig cells (including Star, Cyp11a1, Cyp17a1, Hsd3b2, Hsd17b3, Sf1, positive-Nur77, and negative-Arr19) also exhibited 24-h rhythmic expression patterns (Chen et al., 2017; Gao et al., 2022; Pavlovic et al., 2022), And the circadian clock system was involved to the process of bisphenol A (Li et al., 2021) and zearalenone (Zhao et al., 2021) reducing testosterone production. which indicates a crucial role of the circadian clock in testosterone production. Moreover, disturbances in sleep homeostasis are often accompanied by increased activity of the hypothalamic-pituitary-adrenal (HPA) axis, leading to elevated circulating levels of stress hormones (e.g., cortisol in humans, corticosterone in rodents). Elevated corticosteroid levels lead to decreased testosterone production (Gao et al., 2002; Nollet et al., 2020; Liu and Reddy, 2022). These findings are consistent with the results of the present study.

However, there have been conflicting findings regarding the association between sleep duration and serum testosterone levels. Animal studies have shown that chronic sleep restriction causes a decrease in testosterone level in experimental rats (Chen et al., 2020). Two other studies of healthy adult men found the same results (Leproult and Van Cauter, 2011; Alvarenga et al., 2015). In contrast, some studies have found that sleep duration does not change the concentration of serum testosterone (Chen et al., 2016), whereas others have found that insufficient sleep significantly increases the concentration of serum testosterone (Siervo et al., 2019).

Similarly, the effects of sleep-related factors on sperm quality remain undefined. A survey of 953 young Danish men found a negative U-shaped association between sleep quality and sperm concentration, total sperm count, and normal sperm morphology, with men with higher and lower sleep scores having significantly lower sperm parameters than controls (Jensen et al., 2013). A similar study among Chinese students confirmed a negative U-shaped association between sleep duration and sperm count (Chen et al., 2016). Additionally, domestic and foreign researchers have found that late sleep is associated with decreased sperm quality in studies investigating sleep chronotypes and sperm parameters (Liu et al., 2017; Hvidt et al., 2020). In contrast, some researchers have found no significant association between sleep duration and sperm parameters (Wogatzky et al., 2012; Pokhrel et al., 2019), and no evidence of a correlation between shift work and sperm parameters has been found (Eisenberg et al., 2015).

We found that most of the previous studies were cross-sectional, retrospective, or prospective cohort studies. Because of their observational nature, they could not overcome the influence of unmeasured confounding factors on the results and had the disadvantage of a small sample size. In addition, we assessed sleep-related parameters mainly in the form of self-reported questionnaires, which carries the risk of subjective evaluation, and there is heterogeneity in the methods of assessing sleep characteristics between different studies; therefore, it is difficult to compare results between different studies. Finally, none of the studies accurately determined the periodicity or frequency of sleep-related traits. Therefore, there are inconsistencies in the results of previous studies regarding the association between sleep-related traits and male fertility.

To the best of our knowledge, this is the first study to explore the association between sleep-related traits and male fertility at the genetic level. Second, the MR design reduced the likelihood of confounding factors and other contributors to the observed bias. Third, all instrumental variables used were derived from publicly-available GWAS with substantial data, providing statistical validity for assessing sleep-related traits associated with male fertility.

This study had some limitations. First, the GWAS focused primarily on individuals of European ancestry, which may limit how our findings can be extended to other ethnic groups. Second, the sleep-related characteristics selected were all based on self-reported results, which inevitably led to subjective bias. Next, the sleep-related summary data used in our MR analysis were not stratified by sex, which may have implications for association studies on male fertility. Finally, our analysis has limited power and may therefore lead to false-negative results. Subsequent large-scale epidemiological cohort studies are necessary to determine more accurate associations.

In conclusion,genetically-predicted chronotype is associated with bioavailable testosterone levels. Therefore, healthcare providers may recommend men of childbearing age who are ready for pregnancy to pay attention to sleep according to the human biological clock to reduce the risk of male infertility.

## Data Availability

The original contributions presented in the study are included in the article/Supplementary Material, further inquiries can be directed to the corresponding author.
