# Supplementary material for: Association of sleep traits with male fertility: a two-sample Mendelian randomization study
Source: Front Genet. 2024 Feb 22;15:1353438. doi: 10.3389/fgene.2024.1353438 (PMC10917924; doi:10.3389/fgene.2024.1353438)
Supplement: Supplementary file 1 [file Table1.DOCX]

Supplementary Figure S1: Associations of genetically predicted the sleep characteristics with male infertility.

| **Exposure** | **MR Egger** | | **Weighted median** | | **Simple mode** | | **Weighted mode** | |
| --- | --- | --- | --- | --- | --- | --- | --- | --- |
|  | **OR(95%CI)** | P | **OR(95%CI)** | **P** | **OR(95%CI)** | **P** | **OR(95%CI)** | **P** |
| Chronotype | 0.53(0.05-5.24) | 0.588 | 1.30(0.46-3.64) | 0.621 | 4.84(0.15-155.62) | 0.375 | 3.90(0.12-124.27) | 0.442 |
| Sleep duration | 0.27(0.001-57.17) | 0.635 | 0.78(0.11-5.38) | 0.804 | 7.41(0.07-797.34) | 0.405 | 0.3(0.01-11.44) | 0.517 |
| Insomnia | 0.24(0.0006-88.40) | 0.637 | 0.21(0.01-3.58) | 0.279 | 0.10(0.0005-18.14) | 0.394 | 0.18(0.004-6.82) | 0.36 |
| Snoring | 685.05(0.0002-1.81E+09) | 0.392 | 1.39(0.02-8.20E+01) | 0.874 | 4.35(0.0003-5.14E+04) | 0.76 | 2.33(0.0006-8.75E+03) | 0.841 |
| Dozing | 0.53(9.34E-07-295686.43) | 0.925 | 5.82(9.97E-02-339.57) | 0.396 | 2.26(1.18E-03-4308.73) | 0.834 | 3.39(3.61E-03-3183.40) | 0.729 |
| Daytime nap | 2.28（0.02-315.55） | 0.744 | 4.26（0.56-32.26） | 0.161 | 5.40（0.04-744.95） | 0.504 | 6.72（0.12-380.49） | 0.357 |
| oversleepers | 1.98（0.20-20.13） | 0.567 | 1.27（0.15-10.78） | 0.827 | 0.32（0.0004-214.09） | 0.735 | 1.35（0.18-10.27） | 0.774 |
| undersleepers | 0.83（0.008-81.61） | 0.938 | 8.06（0.28-229.22） | 0.222 | 20.57（0.04-9449.72） | 0.343 | 14.19（0.11-1878.72） | 0.298 |

CI, confidence interval; OR, odds ratio.

Supplementary Figure S2: Associations of genetically predicted the sleep characteristics with the Abnormal sperm.

| **Exposure** | **MR Egger** | | **Weighted median** | | **Simple mode** | | **Weighted mode** | |
| --- | --- | --- | --- | --- | --- | --- | --- | --- |
|  | **OR(95%CI)** | **P** | **OR(95%CI)** | **P** | **OR(95%CI)** | **P** | **OR(95%CI)** | **P** |
| Chronotype | 0.24(0.04-1.37) | 0.111 | 0.72(0.31-1.65) | 0.441 | 0.73(0.08-6.45) | 0.774 | 0.58(0.09-3.83) | 0.57 |
| Sleep duration | 0.02(0.0002-3.06) | 0.137 | 0.43(0.07-2.55) | 0.355 | 0.63(0.01-41.00) | 0.83 | 0.45(0.02-10.54) | 0.621 |
| Insomnia | 0.58(0.006-51.34) | 0.801 | 1.59(0.17-15.23) | 0.687 | 9.84(0.09-1063.68) | 0.345 | 3.56(0.1394.32) | 0.453 |
| Snoring | 19197.91(0.10-3.53E+09) | 0.119 | 5.33(0.22-1.28E+02) | 0.302 | 2.24(0.002-2.22E+03) | 0.82 | 2.68(0.004-1.61E+03) | 0.764 |
| Dozing | 0.01(7.90E-08-757.59) | 0.414 | 1.45(3.73E-02-56.01) | 0.843 | 1.99(9.26E-04-4260.87) | 0.862 | 1.27(1.15E-03-1397.22) | 0.948 |
| Daytime nap | 0.17(0.002-11.90) | 0.418 | 0.62(0.11-3.54) | 0.591 | 0.55(0.01-32.01) | 0.774 | 0.51(0.02-12.70) | 0.681 |
| oversleepers | 0.72(0.01-45.15) | 0.879 | 0.28(0.01-5.35) | 0.396 | 0.06(0.0002-12.23) | 0.305 | 0.19(0.002-22.05) | 0.504 |
| undersleepers | 2.86(0.38-21.64) | 0.317 | 4.04(0.48-33.91) | 0.198 | 5.24(0.004-6883.11) | 0.654 | 4.34(0.57-32.90) | 0.166 |

CI, confidence interval; OR, odds ratio.

Supplementary Figure S3: Associations of genetically predicted the sleep characteristics with Bioavailable testosterone levels.

| **Exposure** | **MR Egger** | | **Weighted median** | | **Simple mode** | | **Weighted mode** | |
| --- | --- | --- | --- | --- | --- | --- | --- | --- |
|  | **OR(95%CI)** | **P** | **OR(95%CI)** | **P** | **OR(95%CI)** | **P** | **OR(95%CI)** | **P** |
| Chronotype | 1.13(1.00-1.28) | 0.053 | 1.07(1.02-1.11) | 0.002 | 0.95(0.82-1.11) | 0.55 | 1.03(0.92-1.16) | 0.596 |
| Sleep duration | 1.15(0.80-1.64) | 0.461 | 1.04(0.96-1.13) | 0.325 | 0.88(0.72-1.08) | 0.234 | 0.90(0.77-1.05) | 0.195 |
| Insomnia | 0.82(0.52-1.31) | 0.415 | 1.03(0.93-1.14) | 0.526 | 0.99(0.82-1.21) | 0.956 | 1.02(0.86-1.20) | 0.846 |
| Snoring | 1.34(0.27-6.64) | 0.723 | 1.08(0.93-1.26) | 0.316 | 1.17(0.81-1.68) | 0.406 | 1.19(0.83-1.70) | 0.355 |
| Dozing | 0.64(0.26-1.57) | 0.337 | 0.96(0.81-1.14) | 0.658 | 0.99(0.70-1.40 | 0.938 | 0.94(0.70-1.25) | 0.651 |
| Daytime nap | 0.86(0.64-1.17) | 0.341 | 0.98(0.90-1.07) | 0.641 | 1.08(0.86-1.35) | 0.511 | 0.92(0.78-1.09) | 0.322 |
| oversleepers | 1.09(0.87-1.36) | 0.454 | 1.08(0.93-1.25) | 0.295 | 1.12(0.80-1.56) | 0.516 | 1.10(0.80-1.53) | 0.559 |
| undersleepers | 1.02(0.76-1.36) | 0.906 | 1.04(0.90-1.20) | 0.574 | 1.09(0.79-1.50) | 0.615 | 1.07(0.80-1.44) | 0.643 |

CI, confidence interval; OR, odds ratio.
